# Expression of *Arabidopsis Bax Inhibitor‐1* in transgenic sugarcane confers drought tolerance

**DOI:** 10.1111/pbi.12540

**Published:** 2016-02-13

**Authors:** Daniel Alves Ramiro, Danila Montewka Melotto‐Passarin, Mariana de Almeida Barbosa, Flavio dos Santos, Sergio Gregorio Perez Gomez, Nelson Sidnei Massola Júnior, Eric Lam, Helaine Carrer

**Affiliations:** ^1^ Depto. Ciências BiológicasESALQ/Universidade de São PauloPiracicaba‐SPBrazil; ^2^ Centro de Tecnologia CanavieiraBairro Santo AntonioPiracicaba‐SPBrazil; ^3^ Depto. de Fitopatologia e NematologiaESALQ/Universidade de São PauloPiracicaba‐SPBrazil; ^4^ Department of Plant Biology and PathologyRutgers the State University of New JerseyNew BrunswickNJUSA

**Keywords:** ER stress, PCD, tunicamycin, antioxidant enzymes, photosynthesis, *Saccharum officinarum*

## Abstract

The sustainability of global crop production is critically dependent on improving tolerance of crop plants to various types of environmental stress. Thus, identification of genes that confer stress tolerance in crops has become a top priority especially in view of expected changes in global climatic patterns. Drought stress is one of the abiotic stresses that can result in dramatic loss of crop productivity. In this work, we show that transgenic expression of a highly conserved cell death suppressor, *Bax Inhibitor‐1* from *Arabidopsis thaliana* (*AtBI‐1*), can confer increased tolerance of sugarcane plants to long‐term (>20 days) water stress conditions. This robust trait is correlated with an increased tolerance of the transgenic sugarcane plants, especially in the roots, to induction of endoplasmic reticulum (ER) stress by the protein glycosylation inhibitor tunicamycin. Our findings suggest that suppression of ER stress in C_4_ grasses, which include important crops such as sorghum and maize, can be an effective means of conferring improved tolerance to long‐term water deficit. This result could potentially lead to improved resilience and yield of major crops in the world.

## Introduction

The Brazilian sugarcane agroindustry maintains the largest commercial system of energy production from biomass in the world, producing bioethanol and burning the bagasse for electric power (Furtado *et al*., 2011). At present, sugarcane plantations are concentrated in the central‐south region of Brazil, mainly in the State of São Paulo. Further increase in sugarcane production may involve either boosting higher levels of productivity or expansion of the area for sugarcane cultivation, especially with extensive arable land at the central region of Brazil. However, the introduction of sugarcane to the mid‐west region of Brazil faces a set of challenges as this region is known to experience high temperatures and irregular rainfall during the year. To enable sugarcane farming in these areas, plants must carry a number of physiological and morphological traits for increased tolerance to these abiotic stresses.

One of the first responses of plants to water deficit is the production of reactive oxygen species (ROS) (Carvalho, 2008). If moderate, these ROS are thought to mediate induction of defence pathways and acclimation responses that help in the adaption of plant metabolism to a changing environment. However, if the stress is severe and sustained, the heightened production of ROS can result in damage of nucleic acids, proteins, lipids and other cellular components in the plant, as well as initiation of programmed cell death (PCD) (Dat *et al*., 2000). PCD is an integral mechanism of plant, animal and microbes that functions in the self‐destruction of those cells that are damaged by various stress factors (Lam, 2004). In metazoans, the control of cell suicide is defined by a homeostatic balance between anti‐apoptotic proteins (such as BCL‐2, BCL‐X_L_, BFL‐1, MCL‐1 and others) and pro‐apoptotic proteins (such as those belonging to the BAX and BH3 subfamilies) that tightly regulates the induction of ROS signalling, cytochrome c release from mitochondria and activation of DNase and caspase‐like proteases (Watanabe and Lam, 2009). One of the most well‐characterized pro‐apoptotic protein is BAX (BCL‐2 antagonist X), in part due its capacity to interact with and antagonize a pro‐survival protein belonging to the BCL‐2 subfamily (Oltvai *et al*., 1993). BAX protein can localize onto the mitochondrial membrane and when activated induces cytochrome c release, stimulation of caspases and cleavage of essential proteins leading to cell death (Danial and Korsmeyer, 2004). In *Arabidopsis* and tobacco, the overexpression of mammalian *Bax* results in spontaneous cell death that is dependent on mitochondrial localization of the expressed Bax protein (Kawai‐Yamada *et al*., 2001; Lacomme and Santa‐Cruz, 1999). These observations indicate that a conserved mechanism regulates PCD in plants and animals in spite of the fact that structural homologues of BCL‐2 related proteins are not present in sequenced plant genomes. In yeast, several enzymes that are known to be induced during oxidative stress were isolated from BAX survival screens with plant cDNA expression libraries, suggesting that ROS production plays an important role in BAX‐dependent cell death (Kampranis *et al*., 2000; Moon *et al*., 2002). Similar to apoptosis in animals, plant cell death caused by heterologous expression of mammalian *Bax* is preceded by H_2_O_2_ production (Yoshinaga *et al*., 2005).

The gene *Bax inhibitor 1* (*BI‐1*), an anti‐cell death gene that is conserved in animal and plant genomes, was first isolated from a human cDNA library based on its ability to block cell death mediated by *Bax* in yeast (Xu and Reed, 1998). Contrary to BCL‐2 protein that can physically interact with BAX, BI‐1 acts downstream in the signalling pathway that leads to PCD (Baek *et al*., 2004; Xu and Reed, 1998). In plants, *BI‐1‐*like genes have been isolated from several species and their predicted amino acid sequences showed a high level of homology with the human BI‐1 protein (Kawai *et al*., 1999; Watanabe and Lam, 2009). Despite the homology between *B1‐1* genes, members of BCL‐2 family proteins are not present in plants. However, overexpression of *Arabidopsis BI‐1* (*AtBI‐1*) suppresses cell death induced by the ectopic expression of mammalian *Bax* in yeast (Kawai *et al*., 1999). Similarly, cell death was reduced in transgenic *Arabidopsis* expressing *Bax* when retransformed with *AtBI‐1*, providing a functional validation of the gene *in planta* (Kawai‐Yamada *et al*., 2001). The *AtBI‐1* gene encodes a cytoprotective protein located in the endoplasmic reticulum (ER) membrane that is induced during senescence as well as under various biotic and abiotic stress situations, and in these conditions modulates the activation of PCD (Watanabe and Lam, 2009). Ihara‐Ohori *et al*. (2007) demonstrated an interaction between *AtBI‐1* and calmodulin (CaM), one of the most conserved sensors of Ca^+2^ in eukaryotes. CaM proteins directly bind to Ca^2+^‐ATPases in the ER, indicating a possible role for *AtBI‐1* in calcium homeostasis through its interaction with CaM (Ishikawa *et al*., 2011). Calcium flux from the ER to the cytoplasm of the cell is one of the earliest known events that is activated by oxidative stress (Ermak and Davies, 2002), while the capacity of *AtBI‐1* in the suppression of cell death was abolished in yeast mutants deficient in ion transporter proteins from the ER to the cytoplasm (Ihara‐Ohori *et al*., 2007). In addition, it was demonstrated that *Arabidopsis* BI‐1 can interact with sphingolipid fatty acid 2‐hydroxylases (AtFAH) via cytochrome b_5_ (AtCb_5_‐B) to modulate ER stress responses (Nagano *et al*., 2012). These results thus indicate that AtBI‐1 may be a highly conserved cell death modulator that mediates stress signalling from that ER via alterations in Ca^+2^ fluxes as well as redox signalling via modulation of fatty acid metabolism.

Studying transgenic expression of *AtBI‐1* in sugarcane may provide new insights into the role of BI‐1 in regulating ROS signalling and also can help to develop novel sugarcane varieties that exhibit increased tolerance to drought. In this work, we show that transgenic lines of sugarcane expressing *AtBI‐1* display significantly higher tolerance to drought stress. Also, transgenic sugarcane show altered patterns of development, sugar accumulation in leaves and culms, increased tolerance to stress caused by tunicamycin and higher activity of oxidative stress‐related enzymes, indicating that ROS signalling may be affected by ectopic expression of this conserved anti‐cell death gene. Our results showed that cell death modulation through increased expression of BI‐1 could be an attractive approach to improve drought tolerance in sugarcane, an important energy grass for renewable biofuel production.

## Results and discussion

### Overexpression of *AtBI‐1* in sugarcane increased tolerance to hydric restriction

**Figure 1:**
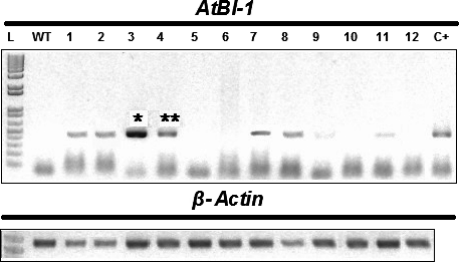
*AtBI‐1* expression in 12 lines of sugarcane transgenic plants. Agarose gel 1% stained with ethidium bromide, showing products of amplification by RT‐PCR from leaves of ScBI (1–12) and WT plants. The plasmid pDM9, harbouring the *AtBI‐1* gene, was used as positive control (C+). β*‐actin* was used as an internal control in the same samples. *ScBI‐3 and **ScBI‐4.

**Figure 2:**
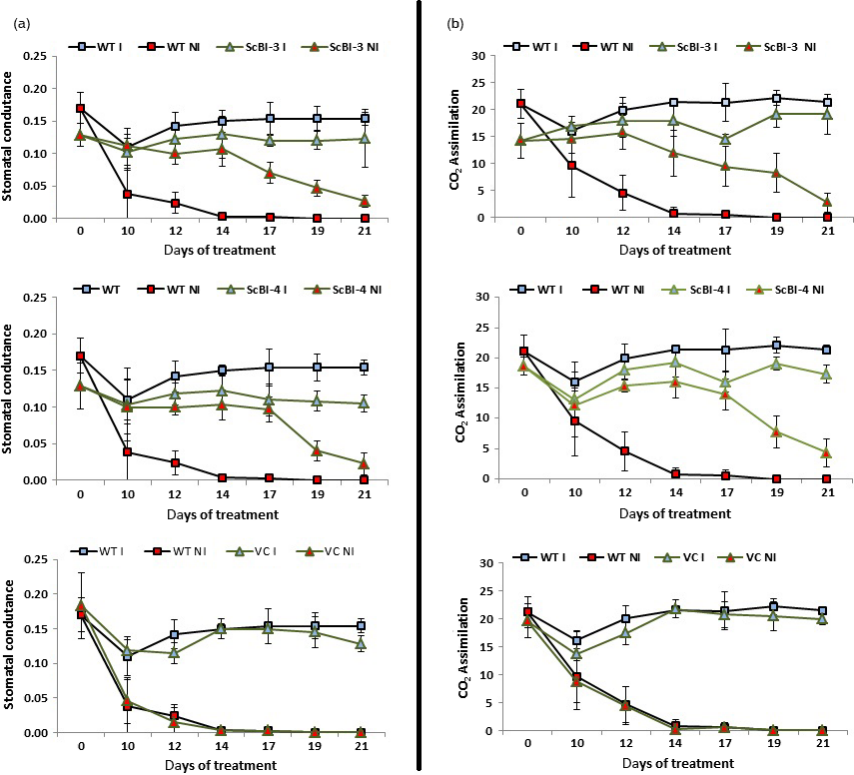
Gas exchange measurements on transgenic sugarcane submitted to drought stress. (a) Stomatal conductance (mol/m^2^/s) and (b) CO_2_ assimilation rate (μmol/m^2^/s) sugarcane adult plants undergoing continuous irrigation (controls) and under drought condition. Plants belonging to ScBI‐3 and ScBI‐4 events have shown *AtBI‐1* gene expression controlled by *Ubi‐1* promoter. Each symbol corresponds to the average value (*n *=* *5) ± SD. I, irrigated; NI, nonirrigated; VC, vector‐only control; WT, wild‐type plants.

**Figure 3:**
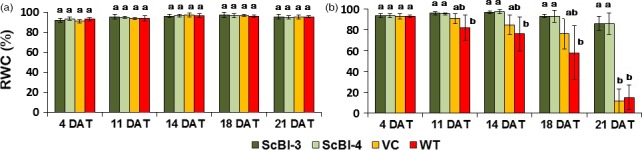
Relative water content (RWC) in leaves of sugarcane in different conditions. Temporal variation of water content in leaves (%) of plants of sugarcane genetically transformed with the gene *AtBI‐1*, in irrigated (a) and nonirrigated (b) condition. Average values (*n *=* *5) ± SD. DAT, days after treatment. Different letters indicate statistic differences between the averages (Tukey, *P *<* *0.05). VC, vector‐only control; WT, wild‐type plants.

The gene *Bax Inhibitor‐1* is implicated to suppress H_2_O_2_‐ and ER‐stress‐induced plant cell death (Kawai‐Yamada *et al*., 2004; Watanabe and Lam, 2008). In model dicot plant species such as *Arabidopsis* and tobacco, overexpression of *BI‐1* has been found to provide increased tolerance to abiotic stresses such as heat and drought stresses as assayed by plant recovery after the stress treatment (Isbat *et al*., 2009; Watanabe and Lam, 2006). In addition, loss‐of‐function *atbi1* mutants in *Arabidopsis* display heightened sensitivity to ER stress induction by the inhibitor tunicamycin, while AtBI‐1 overexpression increased plant tolerance to ER stress (Watanabe and Lam, 2008). Although the mechanisms through which BI‐1 functions to improve stress tolerance in these plant species remain unknown, we wanted to test whether its overexpression in sugarcane, one of the major C_4_ crop plants in the world, can provide improved performance under water stress conditions as well as to quantify other physiological and molecular effects that may mediate the effects of BI‐1. To accomplish these objectives, we generated independent transgenic lines of sugarcane (designated as ScBI lines) overexpressing the *Arabidopsis BI‐1* cDNA (*AtBI‐1*). RT‐PCR (Figure 1) and Western blot analysis (Figure S1) showed that *AtBI‐1* was expressed at the mRNA and protein levels in ScBI transgenic lines. Southern blot analysis confirmed the integration of multiple copies of the transgene in all the tested ScBI lines (Figure S2). The two lines that exhibited the highest apparent *AtBI‐1* transcript levels (Figure 1) were evaluated by water restriction treatments in order to characterize their phenotypic responses in different tissues, such as leaves, culms and roots. Under drought condition, plants tend to reduce water loss to the atmosphere (transpiration) and thereby minimize the rate of dehydration. This adaptive response is mediated in part via reduction in stomatal conductance that leads to closure of the stomates, which in turn limits the availability of CO_2_ for photosynthesis (Chaves *et al*., 2002; Flexas *et al*., 2006). To test whether drought stress in sugarcane may be attenuated by At*BI‐1* overexpression, capacity for photosynthesis and gas exchange was thus measured at various time points after exposing sugarcane plants to treatment of hydric restriction (i.e. withholding irrigation). We found that in the two ScBI lines examined, a significant decrease in stomatal conductance (Figure 2a) and CO_2_ assimilation rate (Figure 2b) was apparent only from about 21 DAT (days after treatment start), whereas in the WT and VC (vector‐only control) plants drastic reduction in the gas exchange and photosynthesis rate was observed at about 12 DAT. Consistent with previous studies (Flexas and Medrano, 2002; Ghannoum, 2009), a reduction in stomatal conductance caused by water stress was accompanied by a parallel decrease in the rate of CO_2_ assimilation of all the sugarcane plants studied here. The longer persistence of CO_2_ assimilation capacity in transgenic sugarcane expressing *AtBI‐1* under water stress conditions indicates the maintenance of primary metabolism in these plants for almost twice as many days as the control plants, suggesting that the effects of water limitation on sugarcane can be dramatically delayed by the expression of the *AtBI‐1* gene. Consistent with this interpretation, relative water content (RWC) analysis showed that leaves of ScBI plants can maintain their level of internal water above 80%, even after 21 consecutive days without irrigation (Figure 3b). RWC levels are frequently used as a reference to control for the relative intensity of hydric stress between samples, with values higher than 75% considered to indicate mild or moderate hydric deficit (Flexas and Medrano, 2002). Therefore, the water restriction treatment imposed on sugarcane plants under our experimental conditions resulted in different levels of hydric deficit between the different genotypes tested in parallel: severe for control plants (WT and VC) and moderate to mild for the two lines of transgenic plants expression *AtBI‐1* by 21 DAT.

### Morphological and biochemical changes in sugarcane overexpressing *AtBI‐1*

Previous studies with overexpression of *BI‐1* in *Arabidopsis* and tobacco plants did not report any obvious morphological or developmental changes (Isbat *et al*., 2009; Watanabe and Lam, 2006). In contrast, we found that ScBI plants showed significant differences between the mean height and leaf area index (LAI) compared with WT plants (Table 1). Seven‐month‐old transgenic sugarcanes with *AtBI‐1* overexpression were significantly shorter than WT, whereas the LAI was statistically higher on the ScBI plants, mainly due to the increased width of the leaves (Figure S3). In contrast to the differences observed in plant height and leaf area index, stalk circumference was found to be similar between genotypes (Table 1). Considering entire plants (leaves, stalks and roots), there were no statistical differences in the fresh and dry mass between transgenic, VC and WT plants (Table_1_SuppInfo). As the density of stomata and trichomes may be considered morpho‐physiological parameters that could modulate the plant's ability to adapt to hydric stress (Ennajeh *et al*., 2010), and stomatal size may affect the dynamics of the response (Drake *et al*., 2013), we also examined these properties in the genotypes of sugarcane in our work. However, we did not detect significant difference (*P *<* *0.05) between transgenic and WT plants for stomata density or size, as well as for the density of trichomes on leaves (Figure S4).

**Table 1:** Evaluation of morphological traits of transgenic *BI‐1* sugarcane (lines ScBI‐3 and ScBI‐4), vector control (VC) and wild‐type plants (WT)

	ScBI‐3	ScBI‐4	VC	WT
Heights (cm)	100.21 (±10.3)^b^	64.00 (±5.08)^c^	131.92 (±6.72)^a^	138.33 (±12.9)^a^
SC (cm)	12.21 (±0.67)^a^	12.75 (±2.76)^a^	13.00 (±0.92)^a^	12.65 (±1.52)^a^
LAI (cm^2^)	282.46 (±23.76)^a^	252.59 (±33.64)^a^	187.97 (±32.51)^b^	170.11 (±6.29)^b^

SC, stalk circumference; LAI, leaf area index = length × width × 0.75. Different letters indicate statistical differences among genotypes (*n *=* *5) (Tukey, *P *<* *0.05).

**Figure 4:**
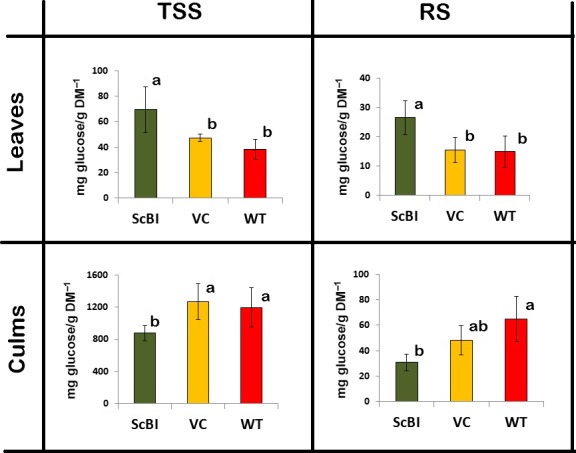
Total soluble sugars (TSS) and reducing sugars (RS) from leaves and culms of sugarcane. Measurements were made on transgenic plants (ScBI) transformed with *AtBI‐1* gene, wild‐type (WT) and vector control plants (VC) under normal conditions of development. The levels are expressed in mg of glucose/g of dry matter. Different letters indicate statistic differences between the averages (*n *=* *5) (Tukey, *P *<* *0.05). DM, dry mass.

In a previous study on gene expression associated with the sugar content of sugarcane, Papini‐Terzi *et al*. (2009) reported that a large number of genes correlated with sucrose accumulation in the culms are associated with stress signalling pathways, such as those activated by ABA and drought. Similar results were also obtained in earlier transcript profiling studies designed to identify genes that are differentially expressed in sugarcane culms during their development (Casu *et al*., 2004). In our present study, we want to ascertain how *AtBI‐1* overexpression may impact sugar accumulation in sugarcane in parallel to the characterization of its response to water stress. We found that the content of total soluble sugars (TSS) and reducing sugars (RS) were statistically higher (82% and 78%, respectively) in leaves of ScBI plants when compared to WT plants (Figure 4). In contrast, the TSS and RS levels in the culms of transgenic plants were statistically lower than those of WT plants (36% for TSS and 110% for RS) (Figure 4). No significant difference was observed between WT and VC plants for TSS or RS in leaves and culms. Results shown here indicate that the overexpression of the *AtBI‐1* gene in sugarcane resulted in alterations in the sugar translocation and accumulation processes in both leaves and culms.

### Effects of *AtBI‐1* overexpression on antioxidant enzyme activities and osmoprotectant levels

**Figure 5:**
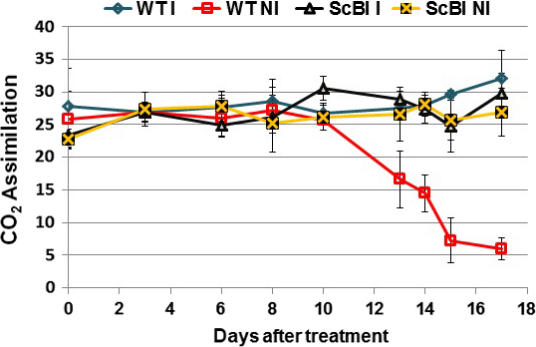
CO_2_ assimilation rates on irrigated and nonirrigated plants. The photosynthetic activity (μmol/m^2^/s) was evaluated on the first totally expanded leaf of 30‐day‐old sugarcane transformed with *AtBI‐1* gene and wild‐type plants (WT) under continuous irrigation (controls) and hydric stress conditions (drought). The levels of drought stress were monitored by measurements of gas exchange until CO_2_ assimilation rate in the nonirrigated WT plants dropped to approximately 5 μmol/m^2^/s. DAT, days after treatment.

**Figure 6:**
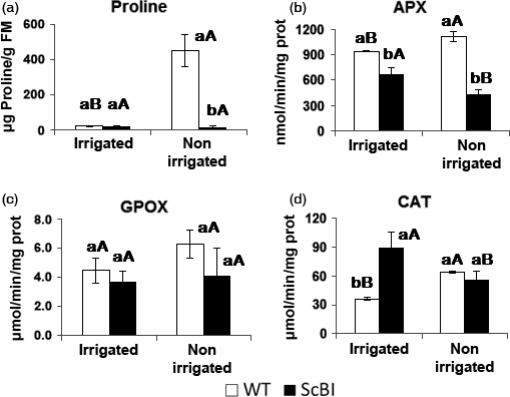
Proline contents and antioxidant enzymes activity of sugarcane plants under drought condition. Proline (a) and antioxidant enzymes ascorbate peroxidase (APX) (b), guaiacol peroxidase (GPOX activity) (c) and catalase (CAT) (d) levels on sugarcane leaves (ScBI) transformed with *Bax inhibitor‐1* gene from Arabidopsis and in wild‐type plants (WT) under drought stress caused by interrupting irrigation for a period of 17 days. Error bars indicate standard deviation of four biological samples. Values for each genotype followed by the same small letter and treatment followed by the same capital letter were not significantly different (Tukey 5%).

In parallel to an increase in sugar accumulation, conserved enzymatic and nonenzymatic mechanisms in sugarcane can also be induced during abiotic stresses in order to protect their cells against the toxic effects of ROS generated under these conditions (Krasensky and Jonak, 2012; Molinari *et al*., 2007). Plants protect their cells by removing or neutralizing ROS through the activation of antioxidant systems. This is often manifested by an increase in enzyme activities such as those of superoxide dismutase (SOD), catalase (CAT), guaiacol peroxidase (GPOX), ascorbate peroxidase (APX) and polyphenol oxidase (PPO), as well as the synthesis and accumulation of osmoprotectants such as proline (Ashraf and Foolad, 2007; Molinari *et al*., 2007; Reddy *et al*., 2004). Increases in the activities of such enzymes and of proline are thus good indicators of oxidative stress response in plants (Reddy *et al*., 2004). In order to establish more precisely the experimental time point at which WT sugarcane plants is likely to be under drought stress, an additional time course experiment was carried out to monitor the stress level indirectly through measuring the decrease in CO_2_ assimilation rate in WT and ScBI sugarcane plants put under hydric stress (Figure 5). At 17 DAT, when the photosynthesis level in WT plants decreased to about 5 μmol/m^2^/s, leaf samples from all the plants in the experiment were collected and used for quantification of enzyme activities for CAT, GPOX and APX, as well as the content of TSS, RS and proline in the same tissues. We found that in the ScBI‐3 and ScBI‐4 plants, oxidative stress markers were not activated by exposure to hydric restriction treatment at 17 DAT, in contrast to significant increases on the level of all oxidative markers that was observed in the WT plants (Figure 6b–d). Similarly, nonirrigated WT plants showed a dramatic increase in the foliar content of proline at 17 DAT, as compared to irrigated plants (Figure 6a). The concentration of proline in irrigated WT plants was 22.4 μg/g of fresh mass, whereas in stressed plants these values rose by about 20‐fold. With ScBI plants, the levels of proline remained low and stable during this experimental condition (20.9 μg/g FM on irrigated plants and 18.5 μg/g FM on nonirrigated plants) (Figure 6a). Interestingly, ScBI irrigated plants showed significantly higher catalase activity, but not that of APX or GPOX, when compared to WT plants before hydric stress was imposed (Figure 6).

**Figure 7:**
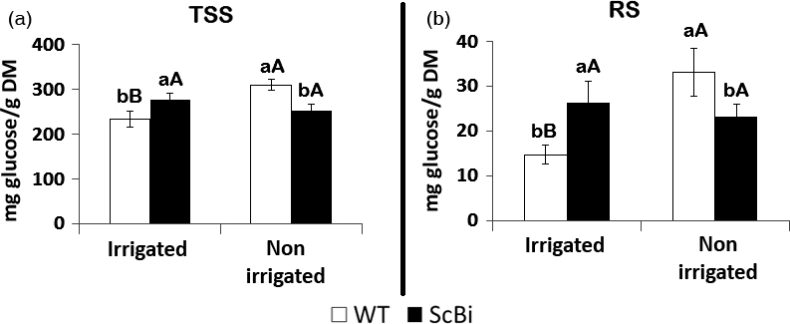
Levels of total soluble sugars (TSS) and reducing sugars (RS) in leaves of stressed sugarcane. Sugarcane transformed with *Bax inhibitor‐1* gene (ScBI) from *Arabidopsis* and wild‐type plants (WT) subjected to hydric stress provoked by the interruption of irrigation during a period of 17 days. Error bars indicate standard deviation of four biological samples. Values for each genotype followed by the same small letter and treatment followed by the same capital letter were not significantly different (Tukey 5%). DM, dry mass.

In addition to quantifying enzyme activities related to oxidative stress, we also measured the level of sugars in sugarcane leaves under drought stress. In response to hydric stress, soluble sugars can accumulate in leaves (Krasensky and Jonak, 2012) and could contribute to the maintenance of the cells' turgor and to the osmoprotection of membranes and proteins (Kaplan and Guy, 2004). Under our assay conditions, we found a significant increase (Tukey test 5%) in the TSS and RS levels in leaves of the WT plants under hydric restriction when compared to the irrigated ones (Figure 7). The hydric stress resulted in an increase of approximately 33% in the levels of TSS (Figure 7a) and 125% (from 14.72 to 33.18 mg glucose/g dry per mass) on the levels of RS (Figure 7b) in WT sugarcane leaves. In contrast, no significant change in leaf TSS and RS content was observed between irrigated and nonirrigated ScBI plants (Figure 7a,b). This data is consistent with the observation that *AtBI‐1* overexpression resulted in a decrease in sensitivity to hydric restriction, and thus, the sugar metabolic pathway in leaves is less affected by the water stress treatment. In sum, our results suggest that the expression of *AtBI‐1* gene was sufficient to increase tolerance of sugarcane to loss of irrigation for more than 2 weeks. The induction of various antioxidant enzymes in WT sugarcane plants under the water stress treatment suggests that ROS management is part of the signal that mediates normal water stress response in this plant species.

### Evaluation of resistance to the brown rust fungus with ScBI plants

Depending on the pathogen lifestyle, overexpression of *BI‐1* can attenuate or improve plant resistance to microbes (reviewed by Ishikawa *et al*., 2011). In theory, one may expect the spread of biotrophic pathogens could be favoured by the cell death suppression that *BI‐1* expression may confer, consistent with the observed decrease in susceptibility of transgenic barley to biotrophic fungal pathogen *Blumeria graminis* f. sp. *hordei* when *HvBI‐1* expression was lowered by RNA interference (Eichmann *et al*., 2010). However, in the wheat—*Puccinia striiformis* f. sp. *tritici* interaction, the suppression of *TaBI‐1* expression in a resistant wheat genotype produced a moderate increase in susceptibility, suggesting that *TaBI‐1* may contribute to wheat resistance to stripe rust (Wang *et al*., 2012).

**Figure 8:**
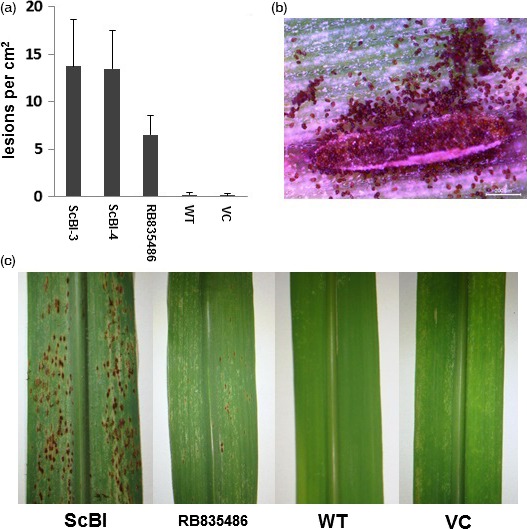
Effect of *AtBI‐1* overexpression on lesion development by *Puccinia melanocephala*. (a) Number of lesions per cm^2^ counted in leaves of ScBI, wild‐type (WT) and vector control plants (VC), 7 days after inoculation (DAI). (b) Sporulating lesion in ScBI leaf at seven DAI. (c) Sugarcane leaves inoculated with brown rust fungus at seven DAI. The variety RB835486 was used as susceptible control.

*Puccinia melanocephala* is the causal agent of brown rust, the major disease of sugarcane in almost all areas in the world where this crop is grown (Dixon *et al*., 2010). As normally observed in most plant–rust interactions, resistance of sugarcane to brown rust is expressed by a posthaustorial hypersensitive response (Oloriz *et al*., 2012). To evaluate the effect of *AtBI‐1* expression on the resistance level of sugarcane to the biotrophic fungus *P. melanocephala*, we inoculated WT, VC and ScBI plants with uredospores from an avirulent race of the fungus. For comparison, we also tested a susceptible sugarcane line, RB835089, in parallel. Our results showed that *AtBI‐1* expression strongly affected the resistance of sugarcane plants to the brown rust fungus (Figure 8). Usually, the latency period (time period elapsed between inoculation and production of spores) of *P. melanocephala* lasts approximately 15 days (DAI). In this study, the latency period of the fungus decreased to 7 DAI on the leaves of the ScBI plants, with large number of spore production (Figure 8b). In addition, the density of pustules was significantly greater in the transgenic lines, even when compared to the susceptible control (Figures 8a). In contrast, WT and VC plants are relatively resistant to the rust fungus (Figure 8c). These results suggest that constitutive *AtBI‐1* expression may suppress resistance against *P. melanocephala* in sugarcane.

### ScBI transgenic sugarcane plants exhibit reduced sensitivity to the ER stress inducer tunicamycin

**Figure 9:**
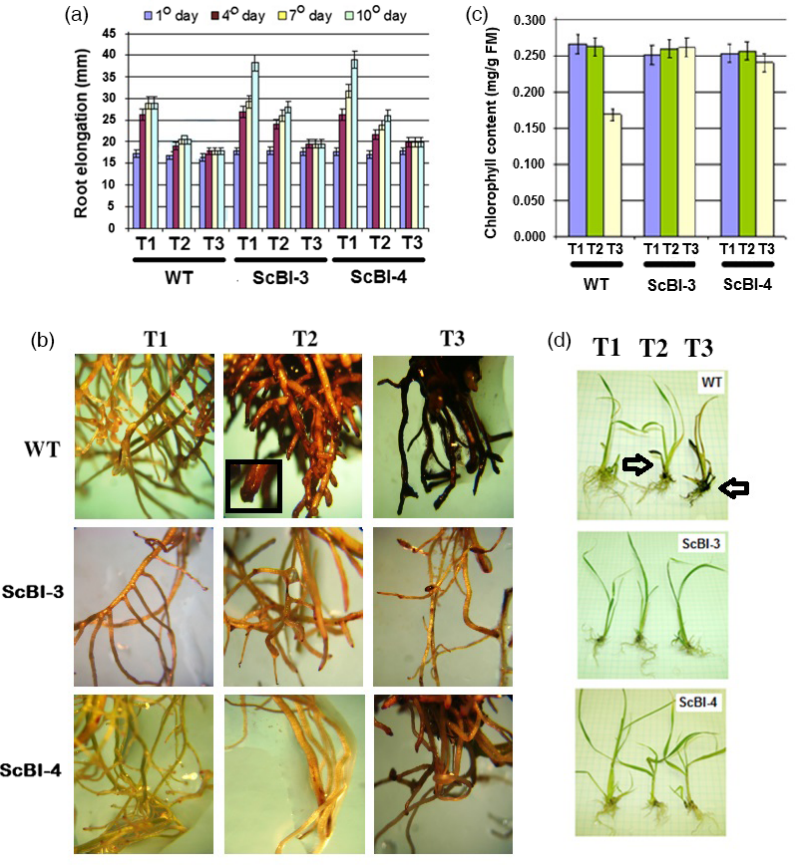
Effect of tunicamycin (TM) on the phenotype of transgenic sugarcane. Plants with 4 weeks of age were incubated in different treatments: T1 (control without TM), T2 (0.5 mg/L TM) and T3 (1.0 mg/L TM) for 10 days. (a) Effect of TM on the sugarcane root elongation of WT plants and transgenic plants SCBI‐3 and ScBI‐4. The vertical length of the root system (mm) was determined at 1st, 4th, 7th and 10th days after the start of the experiment. Error bars indicate standard deviation of 10 biological samples. (b) Phenotype of the sugarcane root system of WT plants and transgenic plants (ScBI‐3 and ScBI‐4) incubated in different dosage of the antibiotic, 10th day after treatments. In the black window: swelling of root tips at T2. (c) Chlorophyll content in WT and transgenic plants (ScBI) growing in liquid MS medium with tunicamycin, after 12 days of incubation. Error bars indicate standard deviation of 10 biological samples. (d) Phenotype of sugarcane growing in liquid MS medium with tunicamycin. Arrows indicates WT plantlets with leaves and roots browning after 12 days of incubation with 1.0 mg/L of TM (T3 condition).

Previous studies with *Arabidopsis* provided evidence for *AtBI‐1* to function in cell death suppression via modulation of the ER stress response pathway's threshold for PCD activation (Watanabe and Lam, 2008). One strong support for this model comes from the suppression of cell death response to the ER stress inducer tunicamycin (TM), which inhibits N‐glycosylation of nascent proteins in the ER. To test whether overexpression of *AtBI‐1* in sugarcane can also suppress cell death activated by ER stress, we tested whether ScBI plants show an attenuated sensitivity towards TM treatment of young sugarcane plantlets grown under hydroponic culture conditions. With WT plantlets, we found that root growth under these conditions became repressed between day 7 and day 10 of culture (Figure 9a). Addition of TM at 0.5 mg/L (T2) and 1.0 mg/L (T3) accelerated this root growth arrest to day 4 or earlier. With two lines of ScBI plants, we observed delayed growth arrest under all three conditions. This difference is especially clear under condition T2 where root growth of WT plantlets stopped between day 4 and day 7, while the ScBI plants continue to grow even after 10 days. In addition to quantifying the effects of TM on root growth arrest, we also observed drastic morphological effects on WT plants after 10 days of TM treatment. As shown in Figure 9b, swelling of root tips at T2, and cell death in the roots at T3 in which the tissues often turn black or dark brown were observed. The ScBI lines did not show these severe morphological changes in roots with only some browning of the tissues observed under the T3 condition (Figure 9b). In addition to the cell death observed in roots upon treatment with TM, we also observed similar effects in the aerial tissues of sugarcane plantlets (Figures 9c,d). After 12 days of incubation with 1.0 mg/L of TM (T3 condition), WT plantlets showed leaf browning and a significant loss of chlorophyll content that suggested activation of cell death. In contrast, little change in chlorophyll content is observed for ScBI‐3 and ScBI‐4 plants that were treated similarly and the plantlets' aerial tissues stayed green even under the T3 condition. These results indicate that heterologous *AtBI‐1* expression resulted in the suppression of ER stress sensitivity in both leaf and root tissues of sugarcane plants.

## Conclusions

The highly conserved *BI‐1* gene is a universal suppressor of cell death, found in the endoplasmic reticulum of metazoan and plants, where it acts to modulate cell death signalling (Ishikawa *et al*., 2011). The highly responsive nature of *AtBI‐1* expression under ER stress indicates that this gene may also have a role in the unfolded protein response's connection to PCD activation. In plants, *BI‐1* expression can be induced by a variety of stress stimuli such as pathogen attack, oxidative stress and heat stress, and its overexpression can suppress the activation of cell death (Huckelhoven, 2004; Watanabe and Lam, 2004). Potentially, these stresses lead to the accumulation of ROS and increase the level of cytosolic calcium in the cell, resulting in activation of cell death (Hofius *et al*., 2007; Lam, 2004). Given its location in the ER, which is the largest reservoir of intracellular calcium and an organelle that is sensitive to external and internal stresses, BI‐1 could act by regulating the concentration of cytosolic calcium and/or redox state. Although the precise mechanism of BI‐1 function remains to be elucidated, previous genetic studies have established the function of *AtBI‐1* as a critical determinant for plant survival under ER stress in *Arabidopsis*. The present studies demonstrate that the heterologous expression of *AtBI‐1* cDNA in sugarcane, a C_4_ monocot species, can attenuate the induction of cell death pathways that are activated during hydric stress or upon chemical‐induced ER stress. The observed increase in tolerance to hydric stress is not necessarily predicted as the anatomy of C_4_ monocots is very different from that of C_3_ dicots, and the role of ER stress response in drought tolerance has not been established. Our results thus provide the first demonstration that overexpression of the *AtBI‐1* gene in a wide range of plant species can likely generate plants that could be highly tolerant to drought, and thus, its deployment can potentially enable cultivation of sugarcane and other crops in more arid environments.

To begin to elucidate the mechanism through which AtBI‐1 overexpression can enhance tolerance to hydric restriction in sugarcane, we quantified soluble sugars and osmoprotectant levels, as well as antioxidant enzyme activities in the different genotypes. Several results are significant. In the first, some of the common antioxidant enzymes and the osmoprotectant proline that are normally induced during hydric restriction in WT plants did not show any dramatic increase with or without the stress treatment. This result shows that AtBI‐1 did not function by ‘pre‐activating’ the water stress response pathway per se. However, the second observation is that sugar levels as well as catalase enzyme activity in the leaves of ScBI‐3 and ScBI‐4 irrigated plants do show a significant increase in the ScBI plants as compared to control plants. Interestingly, the sugar levels in the culms of ScBI‐3 and ScBI‐4 plant lines showed a significant decrease compared to those of the control plants. This suggests that sugar transport and partitioning in sugarcane may be altered by constitutive overexpression of *AtBI‐1*. Although the mechanism of sugar accumulation in the culms of sugarcane has been well studied (McCormick *et al*., 2009; Wang *et al*., 2013), many steps in this process remain unknown. Nevertheless, an extensive microarray study with sugarcane found a strong correlation between the expression of drought stress‐related genes and high sucrose content in the culms of contrasting genotypes (Papini‐Terzi *et al*., 2009). Around 32% of genes known as responsive to drought stress in sugarcane were associated with increased sucrose content in mature and immature internodes of thirty genotypes (Papini‐Terzi *et al*., 2009). Our data presented here suggest that alterations of sugar levels in the ScBI‐3 and ScBI‐4 plants could contribute to the observed improvement in tolerance to hydric stress as manifested by a significant delay in drought‐induced physiological changes.

The higher level of catalase activity observed in ScBI transgenic plants is also intriguing as this enzyme acts on the fast degradation of H_2_O_2_ in the peroxisomes and ER. It is possible that the twofold higher catalase activity present in ScBI transgenic plants before hydric restriction may help to suppress ROS signalling during the early phase of stress onset, providing tolerance to drought and increased susceptibility to rust fungus attack. However, the cause for the observed increase in catalase activity in ScBI transgenic plants remains to be established.

Consistent with previous work in other model plant species, overexpression of *AtBI‐1* in sugarcane was found to suppress the sensitivity to ER stress agents. In both root and leaf tissues, ScBI transgenic plants showed heightened resistance to TM. Consistent with previous indication that ER stress may be induced during heat and drought in other plant models (Liu and Howell, 2010), our results show that suppression of ER‐stress‐mediated cell death in sugarcane during hydric restriction could be a plausible hypothesis for the action of *AtBI‐1* in this crop species. A delay in cell death activation may in turn slow the generation of additional ROS and the subsequent signalling cascade to activate other stress responses in the plant. In this vein, the sensitivity of the sugarcane root tissues to stresses (Figure 9) and the clear ability of *AtBI‐1* overexpression to ameliorate the rapid growth arrest and cell death that we observed may suggest that better root survival in ScBI transgenic plants could be a critical contributor to increased drought tolerance. Finally, our current work also indicated a critical need to determine the optimal tissue and timing‐of‐expression for effective deployment of the *BI‐1* transgene to increase drought tolerance in crop plants. As our results with brown rust illustrated, and consistent with other reports with other phytopathogens (Babaeizad *et al*., 2009), constitutive expression of the *BI‐1* transgene may increase susceptibility to certain disease agents. In addition, the morphological effects on leaf shape in our ScBI plants indicated that *BI‐1* constitutive expression can also cause other subtle changes in plant development. In order to minimize these unintended effects, it would be important to determine whether expression of the *BI‐1* transgene in specific tissues or using drought‐specific promoters can retain its ability to improve drought tolerance in sugarcane. The results from these studies will help future design of optimal AtBI‐1 expression constructs that can optimally suppress ER stress in crop plants both spatially and temporally in order to minimize unintended effects. The SUCEST database of sugarcane transcripts (Vettore *et al*., 2003) and accelerating advances in genome sequencing of sugarcane (Lembke *et al*., 2013) should enable the identification and characterization of tissue‐specific as well as drought‐inducible promoters from this species.

## Experimental procedures

### Plant material and biolistic transformation

Sugarcane is a perennial grass, clonally propagated by sections of a cane stalks with buds. Depending on plant variety and growing conditions, sugarcane is harvested after 12–14 months from an original planting. For in vitro propagation, the apical region of the sugarcane (*Saccharum officinarum*) stem variety RB835089 was collected from 10‐month‐old field‐grown plants. Embryogenic calli were initiated from transverse segments of immature leaves on MS medium (Murashige and Skoog, 1962), supplemented with 5% coconut water, 13.5 μm 2,4‐dichlorophenoxyacetic acid (2,4‐D), 150 mg/L of citric acid, 30 g/L of sucrose (pH 5.8), solidified with 2.3 g/L of Phytagel^®^ (Sigma). Explants were cultured in the dark at 27 °C for 50 days. Four hours before and after bombardment, calli were placed in a culture medium supplemented with 0.4 m mannitol and 0.4 m sorbitol as an osmotic treatment (Vain *et al*., 1993).

Sugarcane transformation was performed by biolistic approach (PDS‐1000/He *Biolistic Particle Delivery System* (Bio‐Rad) using co‐transformation of the plasmid pHA9 (Falco *et al*., 2000) with the vector (pDM9) (Figure S5) constructed by replacing the coding region of the *bar* gene in the vector pAHC20 (Christensen and Quail, 1996) with the coding region of *AtBI‐1* cDNA fused to a V5‐His6 epitope tag at its C‐terminus, isolated from the binary vector pNW166 with *Bam*HI and *Sac*I enzymes (Watanabe and Lam, 2008). The integrity of the pDM9 vector was confirmed by PCR, digestions with restriction enzymes and sequencing.

### RT‐PCR analysis

For RT‐PCR analysis, total RNA was isolated from leaves using TRIzol Reagent (Invitrogen, Carlsbad, CA) according to the manufacturer's instruction. Reverse transcription was performed with the SuperScript First‐Strand Synthesis System for RT‐PCR kit (Invitrogen), using oligo(dT) primers. The sequences of primers for the internal control β*‐actin* were designed based on a clone from the SUCEST project (Vettore *et al*., 2003), which are forward: 5′‐CTTAGGTTGGATCTTGCTGG‐3′ and reverse: 5′‐TTAGAAGCATTTGCTGTGGAC‐3′. PCRs were performed according to the protocol: initial denaturation for 3 min at 95 °C; 30 cycles for 45 s at 95 °C, 45 s at 55 °C, 45 s at 72 °C final; and extension for 10 min at 72 °C. PCRs with same amounts of total cDNA were performed using specific primers for the *AtBI‐1* cDNA (BI‐1 forward: 5′‐GACCTTATGTTGTGCTCTTGT‐3′ and BI‐1 reverse: 5′‐CATTGTAGTCCACACCGAGGT‐3′). As controls, we used the cDNAs from nontransformed sugarcane RB835089 (negative control) and plasmid DNA of the vector pDM9 (positive control).

### Drought tolerance comparative experiments

Transgenic and wild‐type sugarcane plants were transferred to the glasshouse and 60 days later were transferred to 10‐L plastic pots containing a mixture of soil, humus and vermiculite (1 : 1 : 1) (Figure S6). After 30 days of acclimation, two sugarcane transgenic lines (ScBI‐3 and ScBI‐4), control transgenic sugarcane plants carrying only the selection gene [vector control (VC)] and nontransgenic sugarcane plants (WT) were submitted to the following treatments: irrigated (watered with 500 mL of distillated water disposed into the pots every afternoon) and nonirrigated. With the exception of the treatments, all plants were kept under the same conditions and samples at different days were then taken for various comparative assays and measurements.

Measurement of gas exchange was taken by an infrared gas analyser (IRGA) (LCpro+; ADC BioScientific Ltd., Hertfordshire, UK). The leaf CO_2_ assimilation rate (A) and the stomatal conductance (gs) measurements were taken at the middle portion of the first leaf with a visible dewlap (leaf +1), from 10:00 to 14:00 h, with 1500 μmol/m^2^/s photosynthetic photon flux density (PPFD). The levels of drought stress were monitored by measurements of gas exchange rates at 0, 10, 12, 14, 17, 19 and 21 days of treatment (DAT). The experiment was conducted in randomized block delineation, with five replications.

Aiming to compare the effects of *AtBI‐1* overexpression on antioxidant enzyme activities and osmoprotectant levels into different genotypes, we did a new drought experiment using thirty‐day‐old WT and ScBI plants (two plants from each line, ScBI‐3 and ScBI‐4). Sugarcane plantlets were transferred to 10‐L plastic pots containing sterilized soil in glasshouse condition and kept for 30 days in acclimation. After this period, plants were submitted to the same treatments performed in the first experiment until CO_2_ assimilation rate in the nonirrigated WT plants dropped to approximately 5 μmol/m^2^/s. At this moment, leaves and culms of all plants were harvested for biochemical and physiological analyses. The experiment was conducted in randomized block delineation, with four replications.

### Total protein, antioxidant enzymes activity and proline analysis

Protein was extracted from 1.0 g of fresh leaves according to Azevedo *et al*. (1998). Quantification of total soluble protein was performed according to the protocol of Bradford (1976) using BSA (Bovine Serum Albumin) as a standard. Guaiacol peroxidase activity was determined by spectrophotometry according to Matsuno and Uritani (1972). Ascorbate peroxidase was measured according to Nakano and Asada (1981). The activity of catalase was measured according to Kraus *et al*. (1995) as modified by Azevedo *et al*. (1998). The proline content of the leaves was analysed as described by Bates *et al*. (1973).

### Relative water content

The relative water content (RWC) was performed according to the protocol proposed by Barrs and Weatherley (1962). Samples of expanded leaves (1 × 7 cm) were cut with stylus in the portion between the midrib and leaf edge, weighed [fresh weight (FW)], kept in distilled water for 4 h, carefully dried on filter paper and weighed again [turgid weight (TW)]. After weighing, the leaf segments were held at 80 °C for 24 h to obtain the dry weight (DW). The RWC was determined in 4, 11, 14, 18 and 21 DAT using the following formula: RWC = [(FW−DW)/(TW−DW)] × 100.

### Determination of total soluble and reducing sugars

The determination of the levels of total soluble sugars (TSS) was performed by the phenol–sulphuric acid method (Dubois *et al*., 1956). At the end of the experiments for tolerance to drought stress, irrigated and nonirrigated plants had leaves and culms collected and dried at 60 °C. Analyses of colorimetric reactions were performed with a spectrophotometer at 490 nm. The levels of TSS were calculated by interpolation of the absorbance on a standard curve of glucose. The determination of the levels of reducing sugars (RS) was conducted in the same extract obtained for determination of TSS, following the methodology of Somogye and Nelson (Nelson, 1944).

### Evaluation of m**orphological characteristics**

Before the induction of the drought stress, six plants of each genotype were evaluated for morphological parameters such as plant height (m) obtained from the root–shoot transition to the tip of younger leaf (at the top of the plant), stalk circumference (cm) measured in the bottom of the first culm and leaf area index (cm^2^), defined by the formula: LAI = width × length × 0.75, measured in the leaf +3 (Francis *et al*., 1969).

### Evaluation of resistance to the brown rust fungus

For evaluation of resistance to the brown rust fungus, three leaves of four plants chosen randomly from each genotype (WT, VC and ScBI‐3 and ScBI‐4) were inoculated with a fresh uredospore solution (5.3 × 10^4^ spore/ml H_2_O). After inoculation, plants were kept for 2 days in a humid chamber and another 5 days in an incubator (21 °C and photoperiod of 12 h). Thereafter, we evaluated the latent period and the number of pustules per cm^2^. Five plants of the compatible variety RB835486 were used as susceptible control.

### Bioassay of transgenic plants with tunicamycin

Transgenic sugarcane and wild‐type plantlets at 4 weeks of age were incubated with MS liquid medium supplemented with 30 g/L of sucrose (pH 5.8) in three different treatments with the antibiotic tunicamycin (TM), an ER stress inducer, at three different concentrations: T1 (0 mg/L TM), T2 (0.5 mg/L TM) and T3 (1.0 mg/L TM). The plantlets were maintained under 20‐h light/4‐h dark at 27 ± 2 °C. The root length and the morphological changes in leaves and roots of the plants were then measured at 3‐day intervals. After 12 days, the chlorophyll content of the plants was measured according to Lichtenthaler (1987).

### Western blot analysis

See Experimental procedures in Data S1.

### Southern blot analysis

See Experimental procedures in Data S1.

### Measurement of leaf parameters

See Experimental procedures in Data S1.

## Conflict of interest

The authors declare no conflict of interest.

## Supporting information


**Figure S1** Western blot analysis of the AtBI‐1‐V5His6 protein in leaves of transgenic plants. Analysis of immunodetection using anti‐V5 primary antibody and Anti‐IgG of mouse conjugated with alkaline phosphatase second antibody. (a) Approximately 20 μg of total protein from each plant were separated on 12% SDS‐PAGE. (b) Proteins transferred to nitrocellulose membrane incubated with the primary and secondary antibodies. M: Molecular weight markers, C‐: crude extract of the sugarcane WT, columns 1–8: crude extract of transgenic sugarcane. *ScBI‐3 and **ScBI‐4.

**Figure S2** Southern blot analysis for certification of the copy number of AtBI‐1 cDNA in selected transgenic lines. Samples of genomic DNA were digested with restriction enzyme NcoI, generating fragments that contain the transgene. (a) 1% agarose gel (TAE 1× buffer, 3 V/cm) stained with ethidium bromide containing the probe and digested genomic DNA samples of the WT plant and transgenic plants. (b) X‐ray film after exposure of the nylon membrane hybridized with the probe. L: 1 kb plus DNA ladder; S: Probe that is AtBI‐1‐V5His6: T‐Nos with 1000 bp; C‐: nontransformed plant; 1–4: ScBI transgenic lines. *ScBI‐3 and **ScBI‐4.

**Figure S3** Width (cm) of the leaves of transgenic *BI‐1* sugarcane (lines ScBI‐3 and 4), vector control (VC) and wild‐type plants (WT). Different letters indicate statistical differences among genotypes (*n *=* *5) (Tukey, *P *<* *0.01).

**Figure S4** Morphological comparison between leaves of transgenic and wild‐type plants of sugarcane. (a) Stomatal length and width (μm) in leaves of WT and ScBI plants; SL, stomatal length; SW, stomatal width. (b) Stomatal and trichome densities (number per mm^2^) counted in the leaf +3 of five randomly selected plants of each genotype. (c) Nail polish impressions of leaf surface of WT and ScBI plants. Red arrows indicate stomates and yellow arrows indicate trichomes.

**Figure S5** Schematic representation of pDM9 vector with 5764 bp containing the cassette for expression of the cDNA of the gene *AtBI‐1* fused to the V5His_6_ tagged under the control of the *Ubi‐1* promoter and terminator of nopaline synthase.

**Figure S6** Sugarcane drought stress experiments. (a) Plants used to evaluate the physiological parameters of gas exchanges in the first experiment. (b) Transgenic and WT plants used in the second drought experiment. (c) Infra‐Red Gas Analyzer measurement.

**Table S1** Fresh mass (FM) and dry mass (DM) of transgenic *BI‐1* sugarcane (lines ScBI‐3 and 4), vector control (VC) and wild type plants (WT), seven month after planting. The mass index (MI) was calculated by the formula [100 − (DM/FM × 100)]. Equal letters indicate no statistical differences among genotypes (*n *=* *5) (Tukey, *P *<* *0.05).
