# Supplementary figures and images for: Expression of Arabidopsis Bax Inhibitor‐1 in transgenic sugarcane confers drought tolerance
Source: Plant Biotechnol J. 2016 Feb 13;14(9):1826–37. doi: 10.1111/pbi.12540 (PMC5067605; doi:10.1111/pbi.12540)

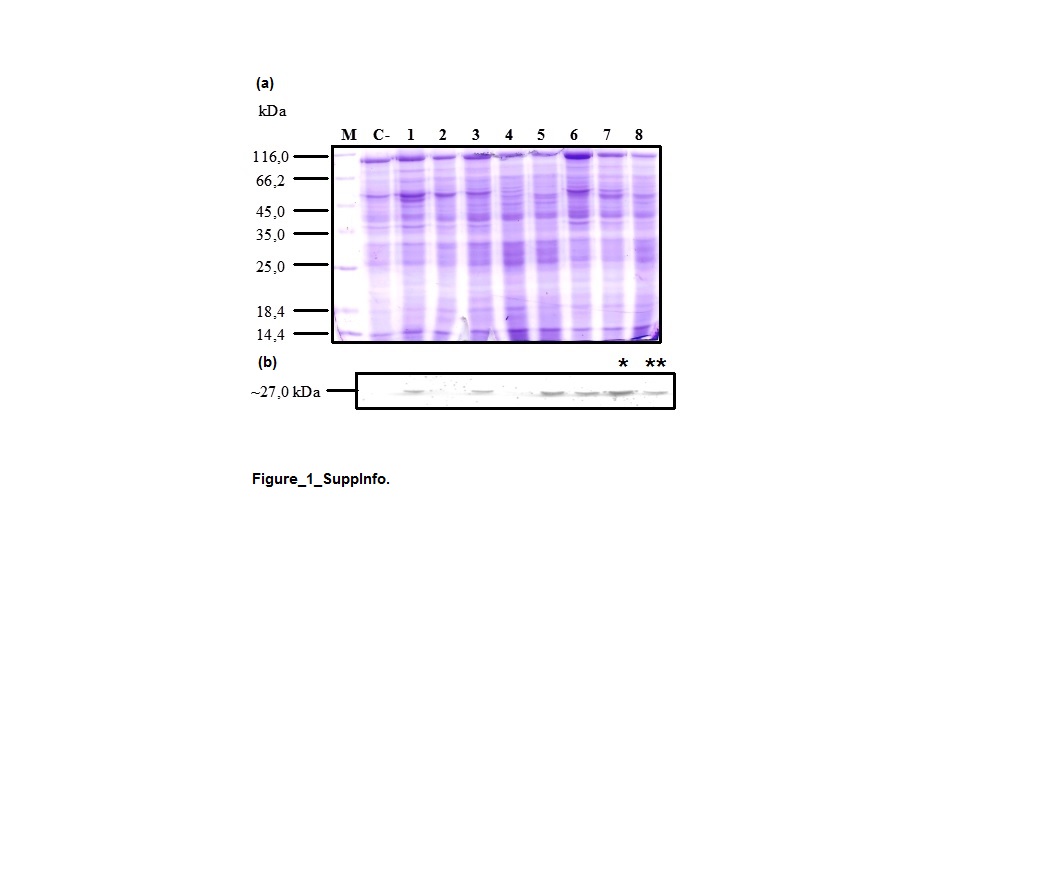

Supplement: Supplementary file 1 — Figure S1 Western blot analysis of the AtBI‐1‐V5His6 protein in leaves of transgenic plants. Analysis of immunodetection using anti‐V5 primary antibody and Anti‐IgG of mouse conjugated with alkaline phosphatase second antibody. (a) Approximately 20 μg of total protein from each plant were separated on 12% SDS‐PAGE. (b) Proteins transferred to nitrocellulose membrane incubated with the primary and secondary antibodies. M: Molecular weight markers, C‐: crude extract of the sugarcane WT, columns 1–8: crude extract of transgenic sugarcane. *ScBI‐3 and **ScBI‐4. [file PBI-14-1826-s004.jpg]

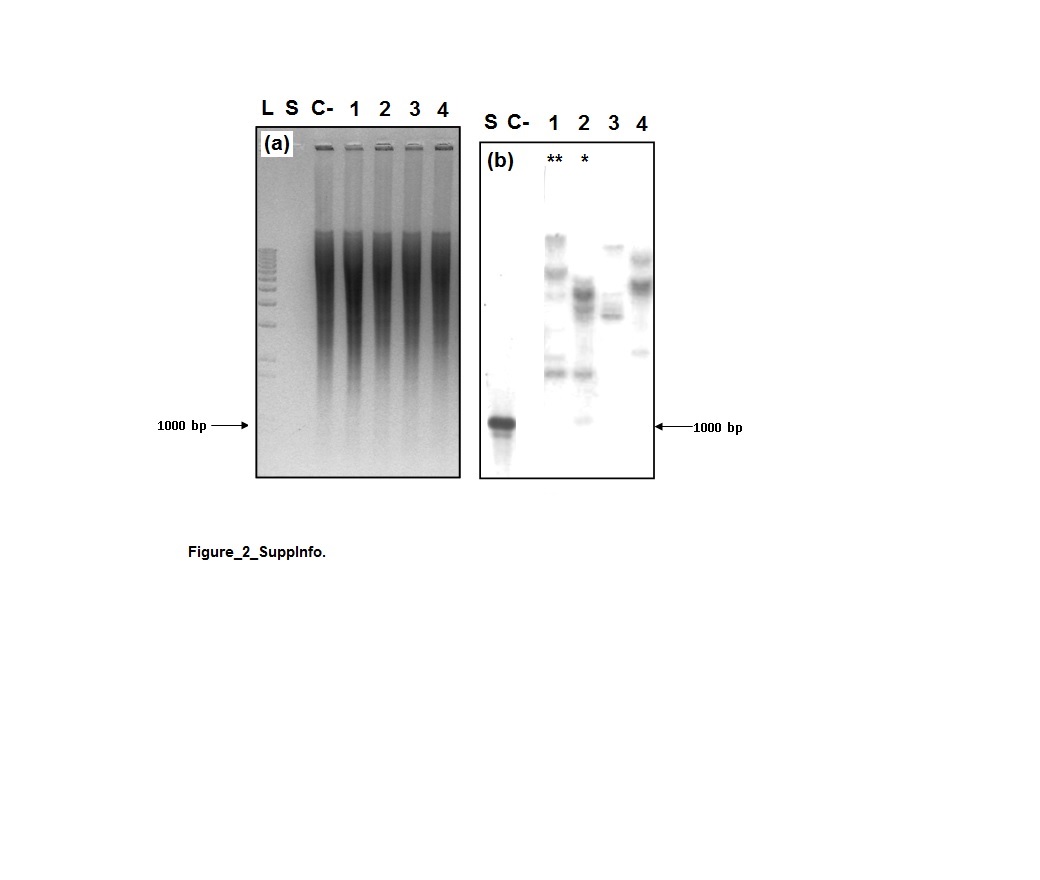

Supplement: Supplementary file 2 — Figure S2 Southern blot analysis for certification of the copy number of AtBI‐1 cDNA in selected transgenic lines. Samples of genomic DNA were digested with restriction enzyme NcoI, generating fragments that contain the transgene. (a) 1% agarose gel (TAE 1× buffer, 3 V/cm) stained with ethidium bromide containing the probe and digested genomic DNA samples of the WT plant and transgenic plants. (b) X‐ray film after exposure of the nylon membrane hybridized with the probe. L: 1 kb plus DNA ladder; S: Probe that is AtBI‐1‐V5His6: T‐Nos with 1000 bp; C‐: nontransformed plant; 1–4: ScBI transgenic lines. *ScBI‐3 and **ScBI‐4. [file PBI-14-1826-s006.jpg]

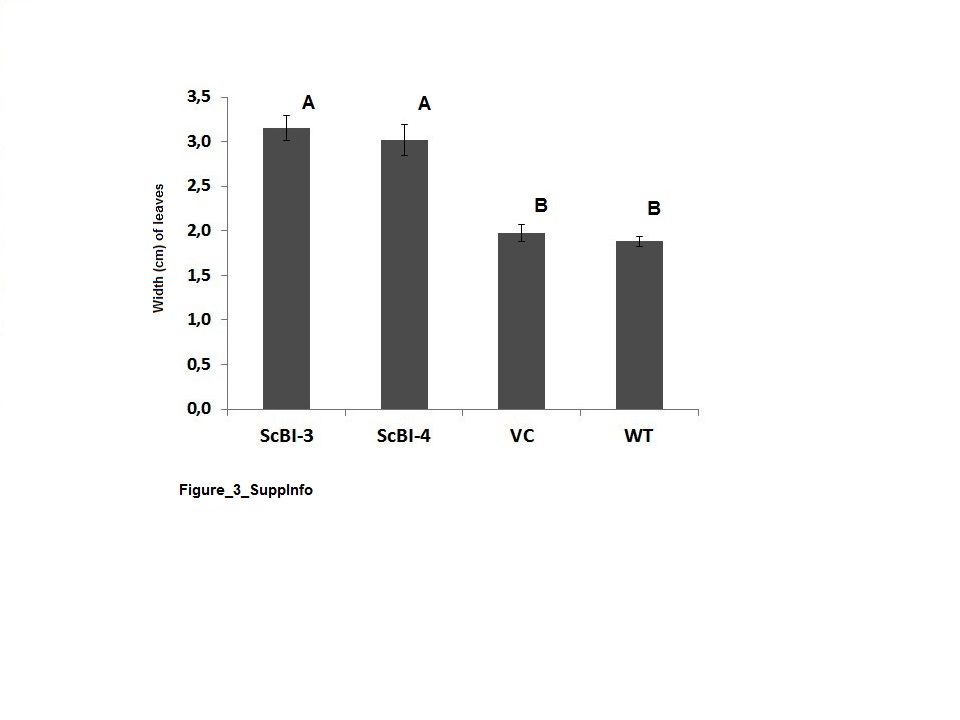

Supplement: Supplementary file 3 — Figure S3 Width (cm) of the leaves of transgenic BI‐1 sugarcane (lines ScBI‐3 and 4), vector control (VC) and wild‐type plants (WT). Different letters indicate statistical differences among genotypes (n = 5) (Tukey, P < 0.01). [file PBI-14-1826-s005.jpg]

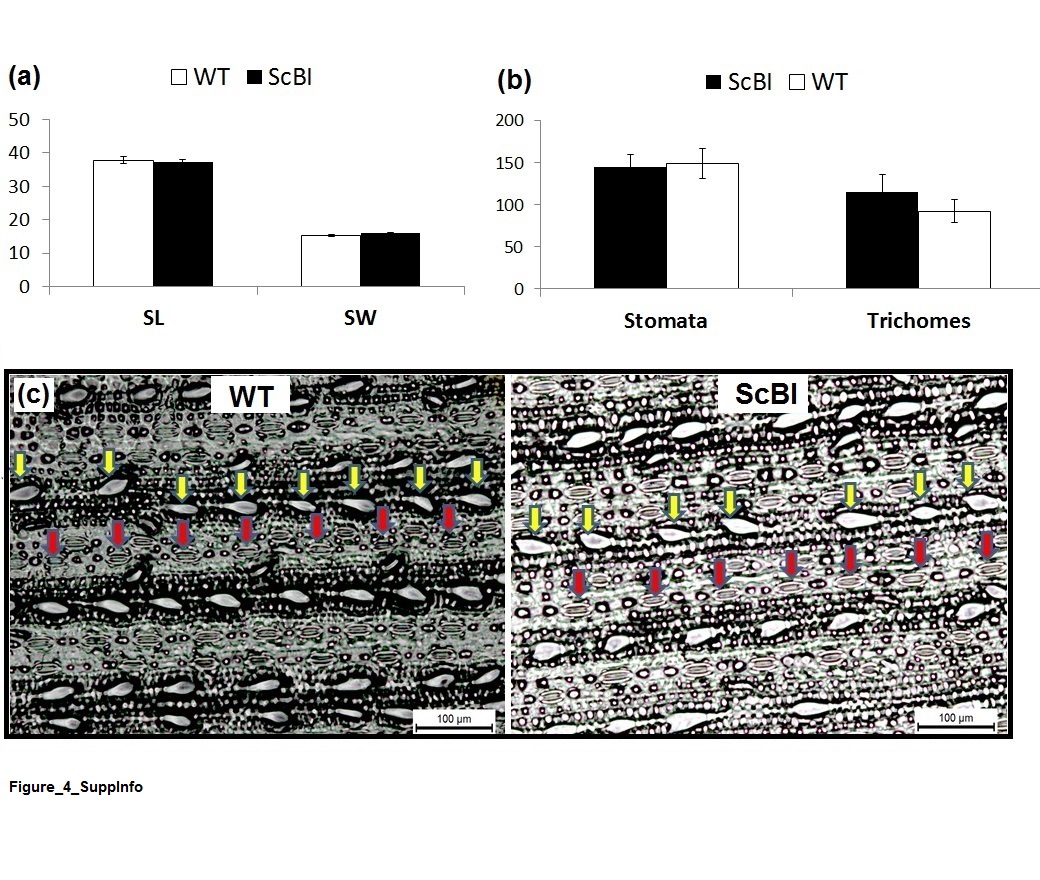

Supplement: Supplementary file 4 — Figure S4 Morphological comparison between leaves of transgenic and wild‐type plants of sugarcane. (a) Stomatal length and width (μm) in leaves of WT and ScBI plants; SL, stomatal length; SW, stomatal width. (b) Stomatal and trichome densities (number per mm2) counted in the leaf +3 of five randomly selected plants of each genotype. (c) Nail polish impressions of leaf surface of WT and ScBI plants. Red arrows indicate stomates and yellow arrows indicate trichomes. [file PBI-14-1826-s007.jpg]

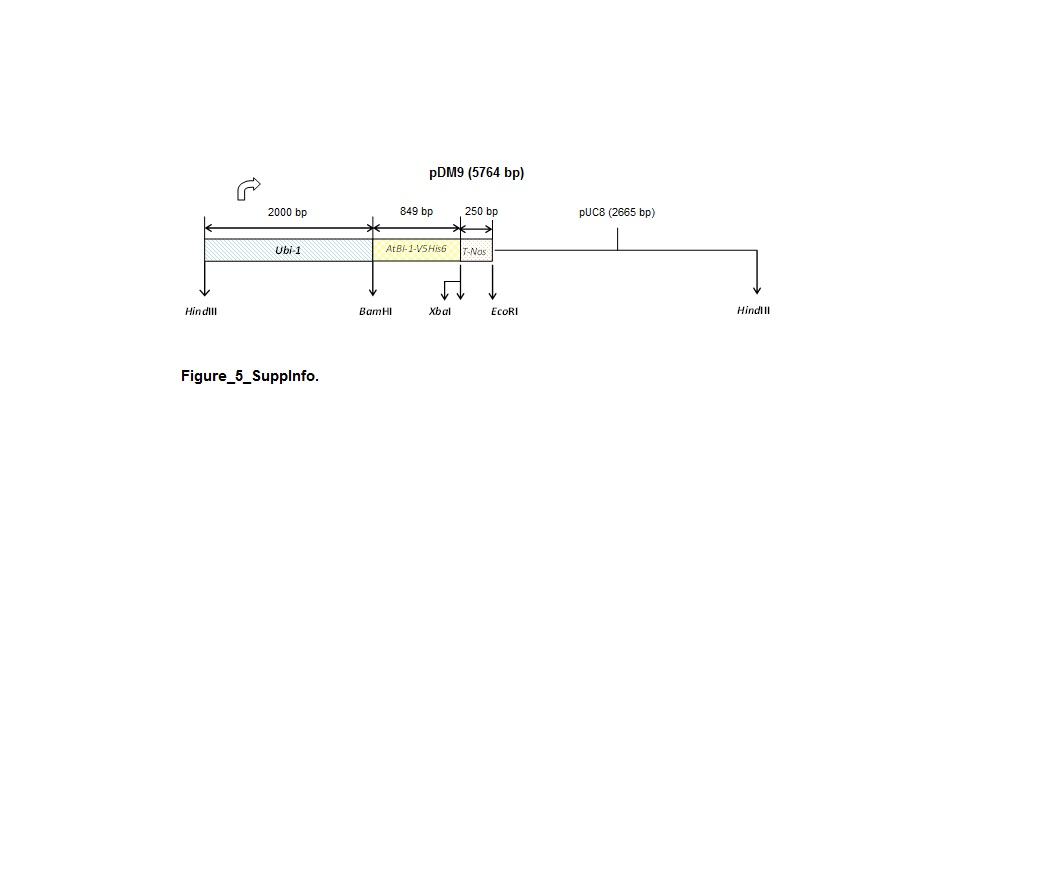

Supplement: Supplementary file 5 — Figure S5 Schematic representation of pDM9 vector with 5764 bp containing the cassette for expression of the cDNA of the gene AtBI‐1 fused to the V5His6 tagged under the control of the Ubi‐1 promoter and terminator of nopaline synthase. [file PBI-14-1826-s001.jpg]

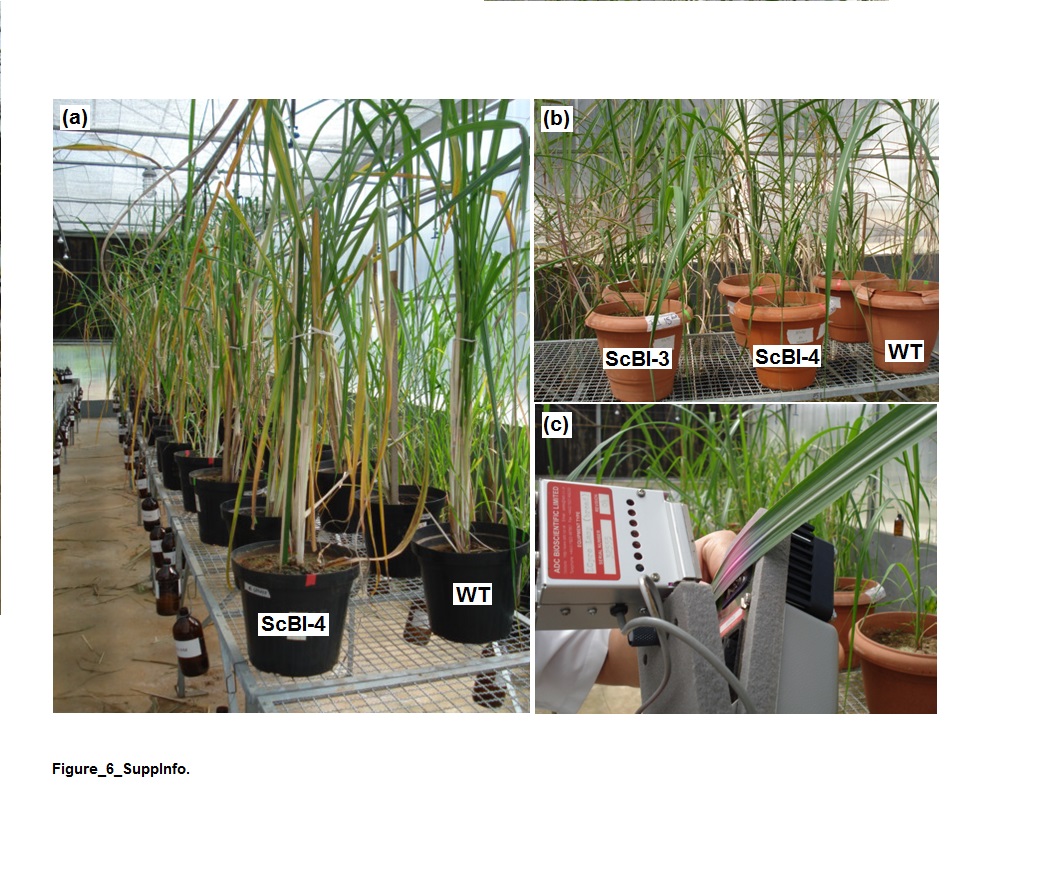

Supplement: Supplementary file 6 — Figure S6 Sugarcane drought stress experiments. (a) Plants used to evaluate the physiological parameters of gas exchanges in the first experiment. (b) Transgenic and WT plants used in the second drought experiment. (c) Infra‐Red Gas Analyzer measurement. [file PBI-14-1826-s002.jpg]
