# Supplementary material for: Expression of Arabidopsis Bax Inhibitor‐1 in transgenic sugarcane confers drought tolerance
Source: Plant Biotechnol J. 2016 Feb 13;14(9):1826–37. doi: 10.1111/pbi.12540 (PMC5067605; doi:10.1111/pbi.12540)
Supplement: Supplementary file 7 — Table S1 Fresh mass (FM) and dry mass (DM) of transgenic BI‐1 sugarcane (lines ScBI‐3 and 4), vector control (VC) and wild type plants (WT), seven month after planting. The mass index (MI) was calculated by the formula [100 − (DM/FM × 100)]. Equal letters indicate no statistical differences among genotypes (n = 5) (Tukey, P < 0.05). [file PBI-14-1826-s003.docx]

Table_1_SuppInfo. Fresh mass (FM) and dry mass (DM) of transgenic *BI-1* sugarcane (lines ScBI-3 and 4), vector control (VC) and wild type plants (WT), seven month after planting. The mass index (MI) was calculated by the formula [100 – (DM/FMx100)]. Equal letters indicate no statistical differences among genotypes (n=5) (Tukey, p<0,05).

|  | **FM** | **DM** | **MI** |  |
| --- | --- | --- | --- | --- |
| **WT** | 219,1 (±58,1) **a** | 34,4 (±17,5) **a** | 85,1 (±3,9) **a** |  |
| **VC** | 169,3 (±38,3) **a** | 25,5 (±6,5) **a** | 84,9 (±0,5) **a** |  |
| **ScBI-3** | 198,7 (±22,0) **a** | 28,3 (±3,5) **a** | 82,8 (±5,9) **a** |  |
| **ScBI-4** | 151,6 (±38,6) **a** | 19,4 (±3,9) **a** | 86,9 (±0,7) **a** |  |
