# Supplementary material for: Expression of Arabidopsis Bax Inhibitor‐1 in transgenic sugarcane confers drought tolerance
Source: Plant Biotechnol J. 2016 Feb 13;14(9):1826–37. doi: 10.1111/pbi.12540 (PMC5067605; doi:10.1111/pbi.12540)
Supplement: Supplementary file 8 — Supplementary Legends [file PBI-14-1826-s008.docx]

**SUPPLEMENTARY INFORMATION**

**Experimental procedures**

**Western blot analysis**

The expression of AtBI-1-V5His6 protein was detected by preparing total protein extracts from leaves by directly grinding the tissues in liquid nitrogen followed by the addition of Laemmli buffer (50 mM Tri-HCl, pH 6.8, 2% SDS, 0.1% bomophenol blue, 10% glycerol, 5% β-mercaptoethanol). Extracts were then incubated on ice for 5 minutes, after heating at 100°C for 5 minutes. The samples were centrifuged at 13.000 x g for 5 minutes at room temperature, and the pellets discarded. Similar amounts of proteins as determined by Coomassie Blue staining were separated on 12% SDS-PAGE gels and blotted onto a nitrocellulose membrane. The blot was then probed with primary antibody anti-V5 (Invitrogen) at a 1:5.000 dilution. The membrane was maintained with the antibody for 4 hours at room temperature under slow and constant agitation, and was subsequently placed at 4°C for 16 hours under the same agitation. The following day the membrane was washed eight times with TTBS buffer (1x) for 8 minutes each, under slowly and constantly stirring at room temperature. Then the membrane was probed with secondary anti-mouse IgG (whole molecule) - alkaline phosphatase (Sigma) at a dilution of 1: 25.000. The membrane was kept under the same stirring for 4 hours and then it was washed eight times for 8 minutes each with buffer TTBS (1x). The detection was performed using a chemiluminescence detection system Lumi-PhosTM WB (Pierce).

**Southern blot analysis**

Southern blot was performed to confirm stable integration of *AtBI-1* cDNA into the transgenic plants. The DNA was extracted and purified as described by Doyle and Doyle (1990). A total of 20 µg DNA was digested with NcoI, separated in agarose gel (1%), and then transferred to a nylon membrane (Hybond-N+, Amersham Biosciences). The AtBI-1-V5His6 probe was prepared by pDM9 digestion with BamHI and EcoRI, and the respective fragment was labeled using Gene ImagesTM Alkphos Direct Labbeling and Detection System kit (Amersham Biosciences). Hybridizations were performed at 55°C and were detected with the CDS-StarTM kit (Amersham Biosciences).

**Measurement of leaf parameters**

Stomatal and trichome densities (number/mm^2^) were measured in nail polish impressions of the abaxial surface of the middle portion of the leaf +3. The impressions were placed on glass slides and viewed under 200X magnification of a light microscope (Leica DM IL LED).

Five WT and SCBI plants were randomly selected and, in each plant, the number of stomates and trichomes were counted in 30 slides. Stomatal length and width were measured in the same slides using the Leica Application Suite (LAS) software (Leica). The experimental design was completely randomized and the data were submitted to analysis of variance (ANOVA).
